# Social Media Use and Psychological and Nutritional Well‐Being in Adults Aged 50 Years and Older: A Cross‐Sectional Study

**DOI:** 10.1002/hsr2.72352

**Published:** 2026-04-15

**Authors:** Kerem Polatoz, Pinar Soysal, Recep Besik, Irem Tanriverdi, Sila Celebi, Ozge Pasin, Lee Smith

**Affiliations:** ^1^ Faculty of Medicine Bezmialem Vakif University Istanbul Turkiye; ^2^ Department of Geriatric Medicine, Faculty of Medicine Bezmialem Vakif University Istanbul Turkiye; ^3^ Department of Biostatistics, Hamidiye Medical Faculty University of Health Sciences Istanbul Turkiye; ^4^ Centre for Health, Performance and Wellbeing Anglia Ruskin University Cambridge UK

**Keywords:** anxiety, depression, loneliness, nutritional status, older adults, social media

## Abstract

**Background and Objectives:**

Although previous studies suggest that social media may influence individuals of different ages, comprehensive and objective research focusing on older adults remains limited. This study aimed to investigate the associations between social media use on the well‐being of older adults using objective assessment methods.

**Methods:**

A cross‐sectional study was conducted with 153 adults aged 50 years and older. Objective social media use was assessed through smartphone screen‐time records covering at least the previous 2 weeks, while participants also reported their estimated daily usage durations. Psychological and nutritional well‐being were evaluated using validated scales: the De Jong Gierveld Loneliness Scale, PHQ‐9 for depression, GAD‐7 for anxiety, and MNA‐SF for nutritional status. Data were analyzed using nonparametric tests, correlation analyses, and multivariable logistic regression.

**Results:**

The mean age of participants was 64.2 years (SD = 9.7), and 43.1% were female. The majority of participants (78.4%) had normal nutritional status, but loneliness (53.6%), depression (62.7%), and anxiety (66.2%) were relatively high. The median daily social media use was 90 min (IQR = 27–178). Older participants spent significantly less time on social media (*p* < 0.001) and tended to overestimate their daily use compared with objective records (*p* < 0.001). When participants were categorized into quartiles according to objectively measured daily social‐media use, higher income levels were also associated with greater use durations (*p* < 0.001). No significant associations were observed between (objective or self‐reported) social media use and loneliness, depression, anxiety, or nutritional status (all *p* > 0.05).

**Conclusions:**

While higher income was associated with greater use, social media engagement was not linked to adverse psychological or nutritional outcomes. These findings suggest that social media use was not significantly associated with adverse psychological or nutritional well‐being among adults aged 50 years and older.

## 1 Introduction

Through the years, social media has become an essential part of most people's daily lives. Originally, social media was created to fulfill the basic communication needs of the modern era, yet over time, social media applications have diversified, gaining a distinct dimension [1]. They are now able to provide us with content on almost any topic of our interest, if not all, in addition to being an irreplaceable way of connecting with people. Generally being easily accessible with a single click, social media continues to strengthen its role in the modern world each day. Recent statistics indicate that 5.24 billion people (63.9% of the global population) are engaged in social media, with 206 million users entering social media in 2025 [2]. According to data from a 2018 study conducted in the United States, 88% of Americans aged 18–29 years use some form of social media application. In the same source, this rate is reported as 64% for those aged 50–64 years, and 37% for the population aged 65 years and older [3]. In a follow‐up study conducted by the same source in 2019, it was observed that this rate had increased to 45% in the 65+ years age group [4]. While social media applications were initially intended for adults to connect and/or engage in business, they are now widely used by varying age groups, ranging from infants to older adults, with an increasingly higher rate of actively engaging in the communities provided by these applications [5].

The effects of social media use on mental health have been investigated by various studies conducted by researchers across varying fields, leading to differing associations associated with social media use. Some studies point to a possible correlation between more hours of social media use and a higher incidence of depression in adolescents [6], while some others suggest a possible link between social media use and lower self‐confidence, increased anxiety, and depression (noting the predominance of its effects on females) [7]. Although the effects of social media are generally thought to be negative outcomes for mental health, there are studies demonstrating that social media might serve as a facilitator of friendships among children [8] and increase creativity among youth [9].

As for the older adults, social media use has been associated with many different impacts as well. As indicated by a systematic review conducted by Guzman et al. [10], multiple studies suggest a positive relationship between social media use and the risk of depression in older adults [11, 12]. There are also studies, such as that of Starvaggi et al. [13], underlining the possibility that social media use has been associated with the spread of misinformation among older adults. Multiple prior research studies suggest that the effects of social media use in older adults can not only be limited to negative ones, though. For instance, Lei et al. [14] suggest that social media use in older adults may reduce loneliness and depressive symptoms, enhancing life satisfaction and quality of life. Similarly, Quinn [15] reported that structured engagement with social media platforms enhanced cognitive processing speed and problem‐solving abilities in older participants. However, while there is a relatively higher number of studies that investigate associations between social media use and its effects on mental health in depth for children and adults in general, there remains a significant gap regarding the elderly.

As it has been suggested by multiple studies, social media use has the potential to affect individuals' daily lives in varying ways, including their eating habits [16]. Although chronically unhealthy eating habits are one of the primary reasons for malnutrition and related nutritional diseases, the association between social media use and the eating habits of older adults remains rather untouched in the geriatric sciences literature. Apart from the very few studies like that of Jiao [17], to our knowledge, most of the studies existing on the topic have been centered around the younger age groups.

There are multiple studies in the literature assessing multiple variables that can be, at least in hypothesis, associated with social media use, like depression [6, 7, 18, 19], anxiety [7], and loneliness [20]. What most of these studies are missing is, though, a stable consensus, as the variability of the findings of the studies is quite high. One reason for this confusion in the literature could be due to the fact that most of the studies conducted on the topic use data gathered from the self‐reports of the participants on their social media use. Among these studies, none have used objective methods such as the assessment of screen‐time logs gathered from the individual cell phones of the participants, except for the studies of Anderl et al. [21], who reported an association between relatively longer hours of smartphone use and relatively worse social connectedness, and Sewall et al. [22], who reported no direct association. It is important to note that none of the studies, to our knowledge, were focused on the geriatric population and that the majority of these studies assessed smartphone use rather than social media use strictly.

Our study highlights the need to re‐examine the accuracy of the findings and the methodologies that were used in previously conducted studies by employing objective methods of screening data and a thorough distinction of usage patterns and usage durations. While there is currently no comprehensive study addressing this issue in Turkiye, the existing international research has predominantly relied on subjective self‐reports rather than measurable outcomes. Through this study, our aim was to address this gap by investigating the association between social media use and the psychological well‐being (loneliness, depression, and anxiety) and nutritional well‐being of older adults with objective and concrete data, thereby providing a more reliable basis for understanding this relationship and supporting future research in the field. To better carry out our analyses and point out the differences in characteristics between those in their middle to late adulthood and the older adults, the minimum age for our study sample was set at 50 years. According to the World Health Organization and United Nations frameworks, older adults are commonly referred to as individuals aged 60 years and above, although it is acknowledged by such frameworks that ageing is a gradual and context‐dependent process, with functional and social transitions often beginning earlier [23]. Therefore, our decision to set the minimum age for our participants at 50 years allowed us to conduct a more detailed examination of social media use patterns across mid‐to‐late adulthood, enabling comparisons with relatively younger participants and highlighting differences in usage characteristics.

## 2 Methods

This study was designed as an observational, cross‐sectional study, using a survey administered face‐to‐face with participants and objective log data obtained from the “Settings” app of each participant enrolled in the study.

The data collection process occurred from February 2025 to July 2025 (~ 7 months). This study was approved by an Institutional Ethics Committee (Approval No. 2024/460). Written informed consent was obtained from all participants.

The inclusion criteria for participants were: being over the age of 50 years, having the Screen‐time feature enabled for at least 2 weeks before the study, and being able to communicate with the surveyor without any major difficulties. Also, participants who had an ongoing mental illness, had been hospitalized within a month, had a history of excessively stressful/traumatic events within a month (e.g., having been affected by a natural disaster, having lost someone special to you, etc.), had undergone a life‐altering acute disease, or had been experiencing any problems that could interfere with their communication with the surveyor were excluded from the study sample. To determine the inclusion status of the participants, the occurrence of any events that may have happened in the past month, possibly altering the participant's daily routine, was questioned, as well as any acute or chronic illnesses they may have had, within their knowledge. To ensure that the participants were not suffering from any illnesses that might have led to their exclusion from the sample, they were asked to provide the names of any medications they had used within the previous month. It was also ensured that participants had not been on vacation during the 2 weeks preceding the survey.

The main measurement tool used for data collection was a survey, which included questions regarding the socioeconomics and demographics of the participants, a question that asked to determine the social media usage patterns of the participants, spaces to fill in the participants' social media usage durations gathered from their cell phones, and any devices they may use. Monthly income was reported in US dollars for standardization purposes.

The means of collecting the social media usage duration was the Screen Time feature present on each individual's cellphone. This feature can be accessed through the following:

- Settings > Screen Time > See All Activity for İOS‐based devices, and
- Settings > Digital Wellbeing & Parental Controls > Dashboard for Android‐based devices (the exact order has slight modifications in each generation/brand).

As mentioned before, having < 2 weeks of data related to social media usage duration was considered among the exclusion criteria. The applications recognized within “social media apps” were those that enable their users to connect with other people in some way. Some of the most commonly used ones were: YouTube, WhatsApp, Facebook, Instagram, X (previously known as “Twitter”), Snapchat, and LinkedIn. The registered data were converted into Mean Daily Usage Duration and used for the analysis.

Questions regarding preserving the accuracy of the log data [such as “Is there anyone who occasionally uses your cellphone and may have interfered with your usage data? (Like a child/grandchild),” and “Which devices do you use to access social media apps? (Cellphone/Tablet/Smart TV)”] were present in the survey. The usage duration data of the participants whose social media usage was separated between more than one device was collected from all of the devices they may have used and registered collectively.

The categorization of the social media usage patterns of the participants was performed according to the typology of social networking site users, developed by Brandtzaeg and Heim [24]. Although this typology was initially intended for categorizing the “Social Networking Site Users,” it was seen fit for classifying the “Social Media Users” due to its high variability and relative accuracy in classifying users. The typology differentiates users into five categories:

- “Sporadics/Occasional Users” are people who use social media apps occasionally, but not frequently. When asked about their social media usage, Sporadics generally reply in phrases such as “It's just not my thing to post videos, etc.”, “You can't count me as a strict social media user, I'm barely online.” or “I just check if I've got any messages on the apps, from time to time.”
- “Lurkers/Passive Users” are users who spend their time passively in social media apps. They mainly indicate that they enjoy “killing time” on social media, rather than taking action to post content or engage with people.
- “Socializers/Social Users” are users whose behavior is characterized by “making new friends” and “engaging in communities.” They mainly spend their time on social media, sending messages to people they know and those they don't, and commenting on others' posts.
- “Debaters” are characterized by being highly involved in discussions taking place in social media communities. The discussions generally follow politics‐related topics, but can vary widely. Debaters tend to describe their social media usage as “sharing their perspectives and knowledge about recent events occurring nationwide and internationally.”
- “Actives/Active Users” are users who engage in almost all kinds of participation activities on social media applications. They are characterized by connecting with people through “sharing content related to and/or unrelated to themselves.” Many Active Users engage in social media applications professionally, working on a salary or a funding basis, although many don't benefit economically from the content they share.

As for the evaluation of the psychological disorders and the nutritional problems the participants may have, four scales were used. The scales were all validated in the Turkish language, and the surveying process was conducted entirely in Turkish as well (see the references for the validation documents of the scales).

- The De Jong Gierveld Loneliness Scale was used to assess the social and emotional loneliness status of the participants. The De Jong Gierveld Loneliness Scale is a scale that is used to assess social and emotional loneliness. It consists of 11 self‐reported items, such as “I experience a sense of emptiness around me” or “I often feel rejected,” which assess emotional loneliness, and items like “I can rely on my friends whenever I need them,” which assess social loneliness [25]. In accordance with the guidelines of the scale, participants' loneliness was categorized as “Low Loneliness (0–2 points),” “Moderate Loneliness (3–8),” and “High Loneliness (9–11).”
- The Patient Health Questionnaire (PHQ‐9) is a self‐reported scale that is used to assess depressive symptoms over the past 2 weeks. It consists of 9 items, such as “Little interest or pleasure in doing things,” “Feeling down, depressed or hopeless,” and “Trouble concentrating on things,” which reflect the emotional, cognitive, and physical aspects of depression [26]. In accordance with the guidelines of the scale, participants' depression was categorized as “Minimal (0–4 points),” “Mild (5–9 points),” “Moderate (10–14 points),” “Moderately Severe (15–19 points),” and “Severe (20–27 points).”
- The General Anxiety Disorder Scale (GAD‐7) is a self‐reported scale to assess the severity of generalized anxiety symptoms. It includes seven items, such as “Feeling very anxious about different issues,” “Being restless, such as to be unable to stand still,” and “Getting easily nervous, angry, or restless,” capturing the emotional and somatic dimensions of anxiety [27]. In accordance with the guidelines of the scale, participants' anxiety was categorized as “Minimal (0–4 points),” “Mild (5–9 points),” “Moderate (10–14 points),” and “Severe (15–21 points).”
- The Mini Nutritional Assessment (MNA) Scale is a test commonly used by clinical practitioners to evaluate the nutritional status of older adults. It assesses multiple domains such as dietary intake, weight loss, mobility, and body mass index to provide a comprehensive overview of a patient's risk for malnutrition [28]. In accordance with the guidelines of the scale, participants' nutritional statuses were categorized as “Healthy Nutrition (24–30 points)” and “At Risk of Nutrition (< 24 points).”

### 2.1 Statistical Analysis

All primary analyses were predefined based on the study objectives. Additional exploratory analyses were conducted to further describe patterns of social media use across subgroups. Descriptive statistics for qualitative variables were presented as frequencies and percentages, while quantitative variables were summarized using mean, median, standard deviation, minimum, and maximum values. Pearson's *χ* ^2^ test and Fisher's exact test were used to evaluate associations between categorical variables.

Binary logistic regression analyses were performed to examine the independent associations between objectively measured daily social media use (categorized into quartiles) and psychosocial outcomes, including loneliness, depression, anxiety, and nutritional risk. Variables that were statistically significant in univariable analyses were subsequently entered into an ordinal logistic regression model to evaluate their independent associations with the outcome. The primary analyses were predefined and focused on examining the association between objectively measured daily social media use and psychosocial and nutritional outcomes. Secondary and exploratory analyses included age‐group comparisons, screen‐time quartile analyses, user typology comparisons, and comparisons between self‐reported and objectively measured social media use.

These exploratory analyses were conducted to provide descriptive and hypothesis‐generating insights. All analyses were performed using IBM SPSS Statistics for Windows, version 28.0 (IBM Corp., Armonk, NY, USA). All statistical tests were two‐sided, and the level of statistical significance was set a priori at *p* < 0.05. A power analysis conducted in January 2025 indicated that a minimum sample size of 153 participants was required; accordingly, the final study sample consisted of 153 adults aged 50 years and older.

Statistical reporting was prepared in accordance with the guidelines for reporting statistics in clinical research in urology and the SAMPL guidelines [29]. Statistical analyses included the Shapiro–Wilk test for assessment of normality, Levene's test for homogeneity of variances, Student's *t*‐test, Mann–Whitney *U* test, Kruskal–Wallis test with Dunn's post hoc comparisons, Pearson's *χ* ^2^ test, Fisher's exact test, Spearman's correlation analysis, and binary logistic regression.

### 2.2 Ethics Approval and Informed Consent

This study was approved by an Institutional Ethics Committee (Approval No. 2024/460). Written informed consent was obtained from all participants.

This study was reported in accordance with the STROBE guidelines for observational studies. As this was a noninterventional, cross‐sectional study, CONSORT guidelines were not applicable.

## 3 Results

A total of 153 participants were included in the study (mean age: 64.2 ± 9.7 years), of whom 43.1% were female. The minimum age among the participants was 50 years, while the maximum age was 90. The majority were aged 50–59 years (38.4%), followed by 60–69 years (33.1%) and ≥ 70 years (29.8%). Most participants lived with their families (90.7%), and overall psychological well‐being was favorable, with relatively low levels of higher than minimal loneliness (53.6%), depression (62.7%), and anxiety (66.2%). Educational attainment was predominantly at the primary‐school level (having been enrolled generally between 7 and 11 years of age), and about one‐third of participants reported a monthly income (see Table 1).

**Table 1:** Sociodemographic, clinical, psychosocial and nutritional characteristics of participants by age group.

Variable	50–59 (*n* = 58)	60–69 (*n* = 50)	70+ (*n* = 45)	Total (*n* = 153)	*p*	Effect size Cramér's *V*
*Gender*					0.795	0.123
Male	37 (63.8%)	28 (56.0%)	22 (48.9%)	87 (56.9%)		
Female	21 (36.2%)	22 (44.0%)	23 (51.1%)	66 (43.1%)		
*Educational background*					< 0.001	0.388
No formal education	0 (0.0%)	2 (4.0%)	4 (8.9%)	6 (3.9%)		
Primary school graduate	11 (19.0%)	20 (40.0%)	23 (51.1%)	54 (35.3%)		
Secondary school	5 (8.6%)	5 (10.0%)	6 (13.3%)	16 (10.5%)		
High school	14 (24.1%)	20 (40.0%)	9 (20.0%)	43 (28.1%)		
University	28 (48.3%)	3 (6.0%)	3 (6.7%)	34 (22.2%)		
*Income level*					< 0.001	0.416
< 600 dollar	8 (14.8%)	15 (32.6%)	25 (55.6%)	48 (33.1%)		
600–1200 dollar	11 (20.4%)	23 (50.0%)	17 (37.8%)	51 (35.2%)		
1200–1800 dollar	16 (29.6%)	3 (6.5%)	3 (6.7%)	22 (15.2%)		
> 1800 dollar	19 (35.2%)	5 (10.9%)	0 (0.0%)	24 (16.6%)		
*BMI groups* a					0.755	0.142
Underweight	9 (16.1%)	11 (22.0%)	7 (15.6%)	27 (17.9%)		
Normal weight	29 (51.8%)	18 (36.0%)	18 (40.0%)	65 (43.0%)		
Overweight	16 (28.6%)	14 (28.0%)	14 (31.1%)	44 (29.1%)		
Obese	4 (3.6%)	7 (14.0%)	5 (11.1%)	16 (10.5%)		
*Living status*					0.096	0.248
Lives alone	3 (5.2%)	6 (12.0%)	7 (15.6%)	16 (10.5%)		
Lives with others	55 (94.8%)	44 (88.0%)	38 (84.4%)	137 (89.5%)		
*Devices used*					0.001	0.289
Smartphone only	43 (74.1%)	37 (74.0%)	31 (68.9%)	111 (72.5%)		
Smartphone + TV	8 (13.8%)	7 (14.0%)	5 (11.1%)	20 (13.1%)		
Smartphone + TV + tablet	3 (5.2%)	2 (4.0%)	3 (6.7%)	8 (5.2%)		
All devices	4 (6.9%)	4 (8.0%)	6 (13.3%)	14 (9.2%)		
*Comorbid diseases*						
Hypertension	33 (56.9%)	25 (50.0%)	24 (53.3%)	82 (53.6%)	0.438	0.276
Diabetes mellitus	10 (17.2%)	8 (16.0%)	7 (15.6%)	25 (16.3%)	0.738	0.200
Chronic kidney disease	2 (3.4%)	2 (4.0%)	3 (6.7%)	7 (4.6%)	0.060	0.173
*Loneliness*					0.824	0.079
Low>	31 (53.4%)	26 (52.0%)	24 (53.3%)	81 (52.9%)		
Moderate	25 (43.1%)	22 (44.0%)	19 (42.2%)	66 (43.1%)		
High	2 (3.4%)	2 (4.0%)	2 (4.4%)	6 (3.9%)		
*Depression*					0.806	0.100
Minimal	36 (62.1%)	31 (62.0%)	29 (64.4%)	96 (62.7%)		
Mild	13 (22.4%)	12 (24.0%)	9 (20.0%)	34 (22.2%)		
Moderate	6 (10.3%)	5 (10.0%)	6 (13.3%)	17 (11.1%)		
Moderately severe	3 (5.2%)	2 (4.0%)	1 (2.2%)	6 (3.9%)		
Severe				0 (0%)		
*Anxiety*					0.762	0.165
Minimal	39 (67.2%)	32 (64.0%)	30 (66.7%)	101 (66.0%)		
Mild	13 (22.4%)	12 (24.0%)	11 (24.4%)	36 (23.5%)		
Moderate	4 (6.9%)	4 (8.0%)	4 (8.9%)	12 (7.8%)		
Severe	2 (3.4%)	2 (4.0%)	0 (0.0%)	4 (2.6%)		
*Nutritional status*					0.562	0.112
Normal nutrition	45 (77.6%)	39 (78.0%)	36 (80.0%)	120 (78.4%)		
At risk of malnutrition	13 (22.4%)	11 (22.0%)	9 (20.0%)	33 (21.6%)		

^a^ The data concerning the body mass index of two participants were missing due to a lack of information provided by the participants.

**Figure 1:**
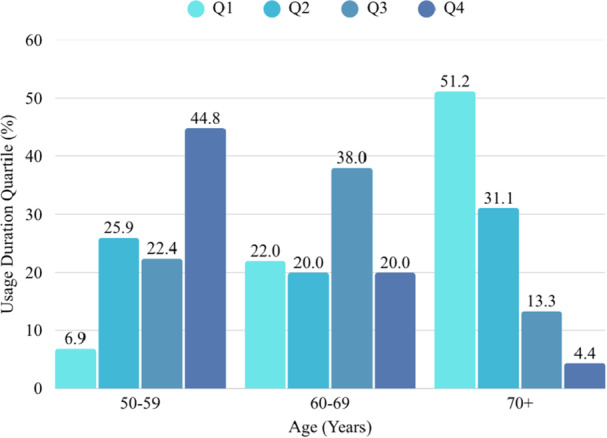
Distribution of social media use duration quartiles across age groups.

Age‐group comparisons revealed several significant differences across sociodemographic and behavioral variables. Educational level (*p* < 0.001), monthly income (*p* < 0.001), number of digital devices used (*p* = 0.001), and social media usage duration (*p* < 0.001) all differed significantly between age groups. Younger participants exhibited higher education and income levels, used more devices, and spent more time on social media. In contrast, gender, BMI, living situation, and comorbidities did not differ significantly among age groups (all *p* > 0.05). Similarly, psychosocial and nutritional variables—including loneliness, depression, anxiety, and nutritional status—were comparable across age categories (all *p* > 0.05; see Table 1 and Figure 1). Regarding social‐media usage types, 10.6% of participants were infrequent users, 41.7% passive users, 13.9% social users, 4.0% debaters, 12.6% active users, and 17.2% nonusers (see Table 2 for the general distribution and the characteristics of each user type). The distribution of usage types differed significantly across age groups (*p* < 0.001). Passive and active social media users were more likely to belong to younger age groups compared with control‐oriented users (as shown in Table 3).

**Table 2:** Distribution of participants by social media user type.

User types	Explanation	Frequency (*n*)
Sporadic users	Those who occasionally spend time on social media applications	16
Passive users	Those who spend most of their time on social media without sharing content or connecting with others. (i.e., scrolling through others' posts)	64
Social users	Those who regard social media applications as a means of connecting with people most commonly through chatting online	21
Debaters	Those who actively participate in forums or in the comment sections of others' posts, frequently engaging in online discussions	6
Active users	Those who actively share content online	19
Control‐oriented users	Individuals who do not use social media applications at all	27

**Table 3:** Ordinal logistic regression analysis of factors associated with age‐group categories.

	*β*	Standard error	*p*	95% confidence interval	
				Lower bound	Upper bound
Occasional users	−0.738	0.711	0.299	−2.132	0.656
Passive users	−2.056	0.592	0.001	−3.215	−0.896
Social users	−1.082	0.673	0.108	−2.400	0.237
Debaters	−1.205	0.984	0.221	−3.134	0.724
Active users	−1.731	0.733	0.018	−3.169	−0.294
Control‐oriented users	Reference				

*Note:* Nagelkerke Pseudo *R* ^2^: 0.482, *p* < 0.001.

When participants were categorized into quartiles according to objectively measured daily social‐media use, several consistent patterns emerged. Mean ages across quartiles (Q1–Q4) progressively decreased from the lowest to the highest usage group, indicating that younger participants spent more time on social media (*p* < 0.001). All user types were significantly associated with higher screen‐time quartiles compared with control‐oriented users (see Table 4). Higher income levels were also associated with greater use durations (*p* < 0.001). However, no significant differences were observed among quartiles in loneliness, depression, anxiety, or nutritional scores (all *p* > 0.05; see Table 5).

**Table 4:** Ordinal logistic regression analysis of factors associated with screen‐time quartiles.

	*β*	Standard error	*p*	95% confidence interval	
				Lower bound	Upper bound
Occasional users	4.783	1.190	< 0.001	2.450	7.116
Passive users	5.182	1.122	< 0.001	2.983	7.381
Social users	5.455	1.168	< 0.001	3.167	7.743
Debaters	5.551	1.371	< 0.001	2.864	8.239
Active users	6.091	1.209	< 0.001	3.722	8.460
Control‐oriented users	Reference				

*Note:* Nagelkerke Pseudo *R* ^2^: 0.557, *p* < 0.001.

**Table 5:** Sociodemographic, clinical, psychosocial, and nutritional characteristics of participants by screen‐time quartiles.

Variable	Q1 (*n* = 38)	Q2 (*n* = 39)	Q3 (*n* = 38)	Q4 (*n* = 38)	Total (*n* = 153)	*p*	Effect size Cramér's *V*
*Gender*						0.795	0.082
Male	19 (50.0%)	23 (59.0%)	22 (57.9%)	23 (60.5%)	87 (56.9%)		
Female	19 (50.0%)	16 (41.0%)	16 (42.1%)	15 (39.5%)	66 (43.1%)		
*Educational background*						< 0.001	0.296
No formal education	6 (15.8%)	0 (0.0%)	0 (0.0%)	0 (0.0%)	6 (3.9%)		
Primary school	16 (42.1%)	15 (38.5%)	16 (42.1%)	7 (18.4%)	54 (35.3%)		
Secondary school	5 (13.2%)	4 (10.3%)	2 (5.3%)	5 (13.2%)	16 (10.5%)		
High school	8 (21.1%)	11 (28.2%)	15 (39.5%)	9 (23.7%)	43 (28.1%)		
University	3 (7.9%)	9 (23.1%)	5 (13.2%)	17 (44.7%)	34 (22.2%)		
*Income level*						< 0.001	0.304
< 600 dollar	24 (64.9%)	8 (21.1%)	8 (23.5%)	8 (22.2%)	48 (33.1%)		
600–1200 dollar	12 (32.4%)	18 (47.4%)	14 (41.2%)	7 (19.4%)	51 (35.2%)		
1200–1800 dollar	0 (0.0%)	8 (21.1%)	5 (14.7%)	9 (25.0%)	22 (15.2%)		
> 1800 dollar	1 (2.7%)	4 (10.5%)	7 (20.6%)	12 (33.3%)	24 (16.6%)		
*BMI groups*						0.755	0.114
Underweight	6 (16.2%)	8 (20.5%)	6 (16.2%)	7 (18.4%)	27 (17.9%)		
Normal weight	16 (43.2%)	13 (33.3%)	18 (48.6%)	18 (47.4%)	65 (43.0%)		
Overweight	9 (24.3%)	15 (38.5%)	9 (24.3%)	11 (28.9%)	44 (29.1%)		
Obese	6 (16.2%)	3 (7.7%)	4 (10.8%)	2 (5.3%)	15 (9.9%)		
*Living status*						0.096	0.210
Lives alone	8 (21.1%)	4 (10.3%)	2 (5.3%)	2 (5.3%)	16 (10.5%)		
Lives with others	30 (78.9%)	35 (89.7%)	36 (94.7%)	36 (94.7%)	137 (89.5%)		
*Devices used*						0.001	0.415
Smartphone only	26 (68.4%)	1 (2.6%)	0 (0.0%)	0 (0.0%)	27 (17.6%)		
Smartphone + TV	5 (13.2%)	19 (48.7%)	23 (60.5%)	17 (44.7%)	64 (41.8%)		
Smartphone + TV + tablet	4 (10.5%)	4 (10.3%)	6 (15.8%)	7 (18.4%)	21 (13.7%)		
All devices	0 (0.0%)	6 (15.4%)	3 (7.9%)	10 (26.3%)	19 (12.4%)		
*Comorbid diseases*							
Hypertension	24 (63.2%)	20 (51.3%)	21 (55.3%)	17 (44.7%)	82 (53.6%)	0.438	0.133
Diabetes mellitus	8 (21.1%)	5 (12.8%)	7 (18.4%)	5 (13.2%)	25 (16.3%)	0.738	0.095
Chronic kidney disease	5 (13.2%)	1 (2.6%)	0 (0.0%)	1 (2.6%)	7 (4.6%)	0.060	0.242
*Loneliness*						0.824	0.098
Low	20 (52.6%)	24 (61.5%)	18 (47.4%)	19 (50.0%)	81 (52.9%)		
Moderate	16 (42.1%)	13 (33.3%)	19 (50.0%)	18 (47.4%)	66 (43.1%)		
High	2 (5.3%)	2 (5.1%)	1 (2.6%)	1 (2.6%)	6 (3.9%)		
*Depression*						0.806	0.111
Minimal	23 (60.5%)	28 (71.8%)	22 (57.9%)	23 (60.5%)	96 (62.7%)		
Mild	9 (23.7%)	6 (15.4%)	8 (21.1%)	11 (28.9%)	34 (22.2%)		
Moderate	4 (10.5%)	3 (7.7%)	7 (18.4%)	3 (7.9%)	17 (11.1%)		
Moderately severe	2 (5.3%)	2 (5.1%)	1 (2.6%)	1 (2.6%)	6 (3.9%)		
Severe					0 (0%)		
*Anxiety*						0.762	0.110
Minimal	25 (65.8%)	30 (76.9%)	24 (63.2%)	22 (57.9%)	101 (66.0%)		
Mild	10 (26.3%)	6 (15.4%)	8 (21.1%)	12 (31.6%)	36 (23.5%)		
Moderate	2 (5.3%)	2 (5.1%)	5 (13.2%)	3 (7.9%)	12 (7.8%)		
Severe	1 (2.6%)	1 (2.6%)	1 (2.6%)	1 (2.6%)	4 (2.6%)		
*Nutritional status*						0.562	0.118
Normal nutrition	27 (71.1%)	33 (84.6%)	30 (78.9%)	30 (78.9%)	120 (78.4%)		
At risk of malnutrition	11 (28.9%)	6 (15.4%)	8 (21.1%)	8 (21.1%)	33 (21.6%)		

Comparison of self‐reported and objectively measured social media use durations revealed significant discrepancies across quartiles (*p* < 0.001; see Table 6). Participants with lower actual use tended to overestimate their daily social‐media time, whereas those with higher usage underestimated it. This pattern suggests that individuals' perceptions of their social‐media engagement are shaped by their actual usage intensity and age.

**Table 6:** Actual and estimated social media use durations across screen‐time quartiles.

Social media use duration quartile groups	Q1	Q2	Q3	Q4	*p*
Actual duration (min)	5.36	57.81	132.72	279.62	< 0.001
Estimated duration (min)	12.06	67.21	104.32	170.42	< 0.001
Variance (min)	−6.7	−9.4	+28.4	+109.2	< 0.001

## 4 Discussion

In the current study, the associations between social media use on psychosocial parameters that reflect the overall psychological well‐being of the participants were investigated through multiple and comprehensive measures. The resulting data demonstrated a significant decrease in the duration of social media use among participants with increasing age. This finding is supported by several studies in the literature [30, 31]. A review conducted by Cotten et al. [31], for instance, noted that although social media use is increasing even among older adults, their rates of use remain lower than those of younger populations. It is nevertheless important to underline that, in the current study, 48.8% of participants aged over 70 years reported using durations higher than 27.1 min daily, while 17.7% reported usage durations higher than 90 min/day. The review also noted that the direction of associations between use and well‐being was inconsistent across studies [31, 32, 33, 34, 35]. The fact that there are several studies reporting results that are in contradiction with each other highlights the complexity of interpreting the findings in relation to factors such as age, usage duration, and usage types. Hence, it is important to consider multiple factors and employ objective methodologies to collect data when investigating possible associations between complex phenomena such as social media use and its effects on individuals' well‐being.

One of the most prominent strengths of our study was that we considered social media user typologies in addition to the duration of social media use. When evaluating the cons and pros of using social media applications, simply taking the use duration is not sufficient, as *how* this time is spent online is generally thought to be a determinant of its effects as well. In the current study, we did not find a significant association between the types of social media use and changes in participants' general characteristics, comorbidity rates, or psychosocial parameters, such as the sense of loneliness, depression, anxiety, and nutritional status. This may be due to the relatively small sample sizes for some of the user types, such as the *debaters* and *the active users*. However, for the user groups such as *passive users* and *social users*, no significant relationships were observed either, even when a sufficient number of participants were met in the analyses. We suggest that future researchers follow a similar approach to evaluating the effects of social media use, with more participants in each user type, to ensure more robust and generalizable results.

On the other hand, there are several studies in the literature that have reported findings that are in disagreement with our findings, and we think the main reason for that is the relatively subjective and nonexhaustive approaches they employed in their studies. For instance, the study conducted by Politte‐Corn et al. [36] in 2023 reported an interesting outcome related to this topic. They reported age‐related differences in the effects of online social support on depressive symptoms. Engaging in online social support was found to be protective of depressive symptoms for adolescents (those aged 10–19) but not for the emerging adults (those aged 19–29). The underlying reason for the difference between the findings of this study and the present study may be that the measurement methods employed in this study are relatively subjective in nature. For instance, their definition of “online social support” based on the positive feedback gathered from the participants may be the underlying reason for the contradiction in the study's findings.

While we did not find a significant association between the types of use and other parameters, it is important to note that the major type of use differed significantly throughout the age groups. The majority of the participants of both the 50–59 years group and the 60–69 years group were concentrated in the passive users category. Similarly, active users and passive users tended to be centered around the younger age groups. The proportion of the participants in the social users category was almost 5 times greater for people between 60 and 69 years than for those between 50 and 59 years, possibly indicating a greater need for socializing than those who are younger than them. Lastly, the participants who were older than 70 years of age were mainly concentrated in the control‐oriented users group.

An important finding to note is that our analyses did not suggest an association between usage duration and psychosocial parameters, in addition to the type of usage. This finding challenges the general assumption regarding the inherently negative impacts of social media use on the psychosocial well‐being of individuals. Although the findings of our study contradicted those of some previous studies [37, 38], we believe that the objective methodology of our study reflects the reality of social media use among older adults more accurately, compared to earlier research.

In addition to psychopathological indicators, there are multiple studies in the literature that have investigated the associations between social media use and broader well‐being dimensions (e.g., life satisfaction, subjective well‐being, etc.) in older adults. For instance, Shamilova and Ayhan [39] reported a positive association between certain motivations for social media use and life satisfaction among older adults, suggesting that the benefits of social media may depend on how individuals engage with these platforms rather than the duration of use alone. Similarly, Sousa et al. [40] demonstrated that social media use among older adults in Portugal was associated with improved social engagement and perceived quality of life, highlighting the potential contribution of digital interaction to overall well‐being. Taken together, these findings indicate that the relationship between social media use and well‐being in older adults is multidimensional and context‐dependent, which may partly explain why no direct association between usage duration and psychosocial outcomes was observed in the present study.

We also did not find a significant association between social media use and nutritional well‐being in older adults, taking into account both the duration and type of social media use. The frequent presence of topics such as healthy living, dieting, and body image on social media platforms significantly influences individuals' eating habits and perceptions of health [41]. These influences can often operate through social comparison, internalization of beauty standards, and the idealization of health‐conscious behaviors, all of which can contribute to changes in eating attitudes [41]. However, the lack of an observed association between social media use and malnutrition in our study may indicate that, in older age, these factors do not substantially influence nutritional behavior. Nevertheless, the association between social media use and nutritional well‐being is still not entirely clear for older adults, as the number of existing studies on the topic is very limited.

Apart from those few studies, like that of Jiao and ours, to our knowledge, the majority of studies are centered around younger populations. This points to the still‐existing gap in studies on this topic.

Unexpectedly, we found a significant association between economic status–indicated by the average monthly income–and social media usage duration of the participants, such that higher income levels were associated with longer durations of social media. Although the underlying reason for this association is not entirely clear, we believe one possible explanation may be that participants with higher income levels often tend to include the use of social media applications as part of their work routine, meaning that their jobs require them to use these applications. For instance, some participants reported that they use social media to advertise the products or services they offer to their customers online. There are also a few studies in the literature that report findings consistent with ours [42, 43], such as the study conducted by Hruska and Maresova [42]. They proposed two possible explanations for this significance: first, individuals with higher income levels tend to have more leisure time compared to those with lower incomes; and second, participants with higher incomes are often those who have integrated their occupations with social media platforms.

In our study, we also observed that the number of devices used decreased with age. This finding may be explained by the general decline in adaptability and biological resilience that accompanies aging. Previous research has shown that advancing age is associated with reduced physiological and cognitive flexibility, resulting in a diminished capacity to adapt to new or unfamiliar conditions [44, 45]. Therefore, older adults may prefer to rely on a familiar device rather than trying new ones. This tendency can also be influenced by factors such as decreased motivation to learn new technologies and the stabilization of daily routines with age. As individuals grow older, they may prioritize convenience and familiarity in technology use, leading to a preference for single‐device use over multiple devices.

Some limitations we had were the cross‐sectional design of our study, the low number of participants in certain user categories (like the Debaters), and the fact that we did not analyze the time spent on each application (like Facebook, YouTube, X, etc.). These factors may have had a role in some of the insignificancies of our analyses. Additionally, we did not record whether the participants used the social media applications as part of their occupational routines or if they were retired or not, which may have contributed to our discussions on the topic. Another factor that could be associated with some insignificancies in the results of our study is the relatively mild and subclinical loneliness, depression, anxiety, and nutrition levels of our participants. Future studies on the topic should consider gathering data such as the specific use dimensions and/or type of content reviewed, and keeping the population size larger and more homogenous regarding user types, as well as the psychosocial and nutritional well‐being groups.

In conclusion, our study employed a different set of methodologies from those in previous research, which we believe contributed considerably to the differences in the findings compared to prior work. When reviewing similar studies in the same field, it was evident that most relied on participants' self‐reports, which may lead to inconsistencies in results, as suggested by Boyle et al. [46] and further supported by the findings of our study. It was one of the primary aims of our study to examine the effects of social media on overall health using objective data rather than relying on self‐reported measures whose reliability may be questionable. In line with this, our findings revealed a significant discrepancy in approaches based on subjective estimations, thereby distinguishing our results from previous studies in the literature that relied on subjective methodologies.

These differences may also be interpreted in the context of sociocultural characteristics of the older Turkish population. Factors such as a relatively stronger sense of intimacy, social bonding, religiosity, and income inequality across different cultural groups may also influence individuals' reliance on such measures for socialization [47, 48]. However, it should be noted that these factors were not directly measured in the present study. Therefore, these interpretations remain speculative and should be considered as potential contextual explanations rather than definitive conclusions. Considering the influence of these numerous factors, the impact of social media use on well‐being may need to be investigated separately for each geographical region.

The findings of our study suggested that there may not be a positive or negative relationship between social media usage and the physical or psychological health of older adults, challenging the general claim that social media use is necessarily harmful. Furthermore, in our study, older age tended to be associated with shorter social media usage duration and a lower number of devices used, while individuals with higher income levels tended to spend more time on social media. These findings provide important insights into the relationship between social media use and factors such as age and socioeconomic status. However, given the cross‐sectional design of the study, these findings should be interpreted as associative rather than causal, and all conclusions should be evaluated at the relational level.

## Author Contributions

Conceptualization: Kerem Polatoz, Pinar Soysal, and Lee Smith. Methodology: Kerem Polatoz, Recep Besik, and Irem Tanriverdi. Formal analysis: Ozge Pasin. Investigation: Kerem Polatoz, Recep Besik, and Sila Celebi. Data curation: Kerem Polatoz and Recep Besik. Resources: Pinar Soysal and Lee Smith. Supervision: Lee Smith. Visualization: Ozge Pasin. Writing – original draft: Kerem Polatoz. Writing – review and editing: all authors. All authors have read and approved the final version of the manuscript.

## Funding

The authors have nothing to report.

## Disclosure

The lead author Pinar Soysal affirms that this manuscript is an honest, accurate, and transparent account of the study being reported; that no important aspects of the study have been omitted; and that any discrepancies from the study as planned (and, if relevant, registered) have been explained.

## Ethics Statement

This study was approved by an Institutional Ethics Committee (Approval No. 2024/460).

## Consent

Written informed consent was obtained from all participants.

## Conflicts of Interest

The authors declare no conflicts of interest.

## Data Availability

The data are not publicly available due to privacy restrictions but are available from the corresponding author on reasonable request. Pinar Soysal had full access to all the data in the study and takes responsibility for the integrity of the data and the accuracy of the data analysis.
